# Progress in Ophthalmology

**Published:** 1898-01-22

**Authors:** 


### PROGRESS IN OPHTHALMOLOGY.

**Functions of the Rods and Cones** *(continued from page* 280).—Molodvoski,[5] in a thesis for the St. Peterburg M.D., shows that the introduction of a pure culture of staphylococcus pyogenes aureus into the conjunctival sac, after an eye operation, varies in the amount of trouble set up, according to the gravity of the operation; thus in the case where the culture was introduced into the conjunctival sac of a rabbit, where the wound was of the cornea or the cornea and lens, only mucopurulent discharge was set up in 24 hours, and was followed by purulent iritis. A wound of a more serious nature produced graver complications; thus when the lens was extracted without loss of vitreous, infection with colonies of the pyogenic coccus caused purulent inflammation of the iris and choroid, while when the extraction of the lens was accompanied by loss of vitreous the whole of the coats of the eye were involved—that is to say, panophthalmitis resulted.

In the cases where the infection was only with the toxin, and the operation was confined to wounding the cornea and lens, simple catarrh of the conjunctiva, with serous inflammation of the iris and ciliary body with hyperæmia of the anterior part of choroid, resulted. When the lens was extracted, with or without loss of vitreous, the serous inflammation involved the choroid. All the wounds infected by the culture became purulent, while those infected by the toxin only healed by first intention. Altogether 65 experiments were made.

**A Method of Obviating Recurrence after Operation for Symblepheron.**—Kenneth Scott[6] mentions a method which he made use of to prevent the exposed surfaces of cornea and conjunctiva uniting after an operation for symblepheron. He everts the eyelid and stitches its ciliary edge to the eyebrow, thus keeping up the eversion until such time as the wound on the globe is covered with epithelium. He recommends sutures of silver wire for this procedure as being pliable, easily fastened by twisting, and, if necessary, unfastened and readjusted. Furthermore, he states that in symblepheron posterius he always cut the flaps from the bulbar conjunctiva, uniting them by buried sutures to the lids, as he finds that the bulbar conjunctiva has greater powers of recuperation than has the conjunctiva on the lids, and that by attaching the flaps to the bare surface of the eyelids any tendency to cicatricial contractions, with its consequent train of ingrowing lashes, &c., is avoided.

**A Case of Diphtheritic Conjunctivitis.**—Mr. Flemming,[7] of University College Hospital, quotes a case of undoubted diphtheritic conjunctivitis which was treated locally with a quinine lotion, gr. iv.-℥i., and in which, although the bacillus diphtheriæ was found in abundance before the use of the lotion, all subsequent examination for the bacillus gave negative results. An injection of anti-toxin was used later on, and the next day it was remarked that the membrane on the conjunctiva was much less marked, the discharge from the eye being distinctly purulent. On the next day the local condition was markedly improved, as scarcely any membrane was left on the lids; later on in this day the child died, in spite of the general treatment. Mr. Flemming draws attention to the fact that no bacilli were found after the application of quinine lotion, and before the anti-toxin was used.

**Subconjunctival Injections.**—As to the[8] utility of subconjunctival injections of bichloride of mercury in various eye troubles, Morton, in the *Practitioner*, quoting Mellinger, gives his conclusions as follows: (1) The beneficial action of subconjunctival injections is due to their stimulating the lymph circulation in the eye; (2) subconjunctival injections of bichloride of mercury 1-2,000 set up an adhesive inflammation, causing an obliteration of the subconjunctival space, and, if repeatedly used, are often accompanied by severe pain; (3) subconjunctival injections of salt solution have the same favourable therapeutic results as the bichloride injections without their severe pain.

**Treatment of the Ordinary Forms of Strabismus.**—Again the same author,[9] quoting Landolt, says that advancement of the opposing rectus should always be preferred to tenotomy of the stronger muscle; that an eye is never over corrected by advancement; while one often sees dire results following tenotomy. Having described the operation in detail, he says: After the operation, both eyes are bandaged for five days at least in divergent strabismus, and for several more days in convergent strabismus, so that there shall be no inducement for the patient to use his two internal recti together. As soon as the advanced muscle or muscles are firmly attached, one eye is left open. It is important, he says, that the patient on whom this operation is performed should be kept in bed, with both eyes bandaged, for the first few days.

**Extraction of Transparent Lens in Extreme Myopia.**—The *American Med.-Surgical Bulletin*[10] reports, in relation to extraction of transparent lens in high myopia: From a total of 400 or 500 cases carefully compared we can draw a certain number of conclusions largely favourable to this intervention; and says, "It is perfectly justifiable in high progressive myopia from 16 D upwards, if there is absence of macular lesions, transparent media, and no amblyopia" (from strabismus or otherwise). They say the operation is not free from a fair percentage of failures, but, even with these, the gravity of complications to be dreaded in unrelieved myopia ought to commend the operation, even at the risk of failure, which, after all, is exceptional, and will become rarer with greater care in antisepsis and in rejection of unsuitable cases.

[5] The Lancet, Sept. 4, 1897. [6] The Lancet, July 31, 1897, page 253. [7] Lancet, October 2, 1897. [8] The Practitioner, Sept., 1897, page 310. [9] Ibid., page 311. [10] American Medico-Surgical Bulletin, Sept. 25, 1897, p. 848.

